# Rational design of transcranial current stimulation (TCS) through mechanistic insights into cortical network dynamics

**DOI:** 10.3389/fnhum.2013.00804

**Published:** 2013-11-26

**Authors:** Flavio Fröhlich, Stephen L. Schmidt

**Affiliations:** ^1^Department of Psychiatry, University of North CarolinaChapel Hill, NC, USA; ^2^Department of Cell Biology and Physiology, University of North CarolinaChapel Hill, NC, USA; ^3^Department of Biomedical Engineering, University of North CarolinaChapel Hill, NC, USA; ^4^Neurobiology Curriculum, University of North CarolinaChapel Hill, NC, USA; ^5^Neuroscience Center, University of North CarolinaChapel Hill, NC, USA

**Keywords:** transcranial current stimulation, electric field, brain stimulation, rational design, optogenetics, feedback control, resonance, cortical oscillation

## Abstract

Transcranial current stimulation (TCS) is a promising method of non-invasive brain stimulation to modulate cortical network dynamics. Preliminary studies have demonstrated the ability of TCS to enhance cognition and reduce symptoms in both neurological and psychiatric illnesses. Despite the encouraging results of these studies, the mechanisms by which TCS and endogenous network dynamics interact remain poorly understood. Here, we propose that the development of the next generation of TCS paradigms with increased efficacy requires such mechanistic understanding of how weak electric fields (EFs) imposed by TCS interact with the nonlinear dynamics of large-scale cortical networks. We highlight key recent advances in the study of the interaction dynamics between TCS and cortical network activity. In particular, we illustrate an interdisciplinary approach that bridges neurobiology and electrical engineering. We discuss the use of (1) hybrid biological-electronic experimental approaches to disentangle feedback interactions; (2) large-scale computer simulations for the study of weak global perturbations imposed by TCS; and (3) optogenetic manipulations informed by dynamic systems theory to probe network dynamics. Together, we here provide the foundation for the use of rational design for the development of the next generation of TCS neurotherapeutics.

## Introduction

Modulating cortical network dynamics with transcranial current stimulation (TCS) has shown promise as a treatment of neurological and psychiatric illnesses (Brunoni et al., 2012; Demirtas-Tatlidede et al., 2013; Floel, 2013) and as an enhancer of cognition in healthy subjects (Hamilton et al., 2011; Kuo and Nitsche, 2012; McKinley et al., 2012). TCS creates a small (subthreshold) change in the membrane voltage of cortical neurons (Jefferys, 1995). The effect of a weak electric field on the membrane voltage depends on the cell morphology, such that large pyramidal cells with extended dendritic trunks are substantially more susceptible to TCS than inhibitory interneurons with more symmetric cell morphologies (Tranchina and Nicholson, 1986; Radman et al., 2009). Importantly, the resulting depolarization induced by TCS is likely limited to about 2 mV, and therefore is insufficient to cause action potential firing in absence of depolarization caused by endogenous network activity. Therefore, any study of TCS will need to include considerations of the ongoing activity during stimulation. In particular, several recent studies have shown that periodic stimulation with alternating current (to mimic transcranial alternating current stimulation (tACS)) enhances endogenous or pharmacologically induced oscillatory activity in slice preparations of cortical tissue. These studies have provided fundamental insights into how TCS interacts with endogenous activity. Here we highlight several recent conceptual and methodological advances that build on this earlier work and together provide the foundation for the rational design of new TCS paradigms.

## Probing Endogenous Electric Fields (EFs) with a hybrid system

**Figure 1:**
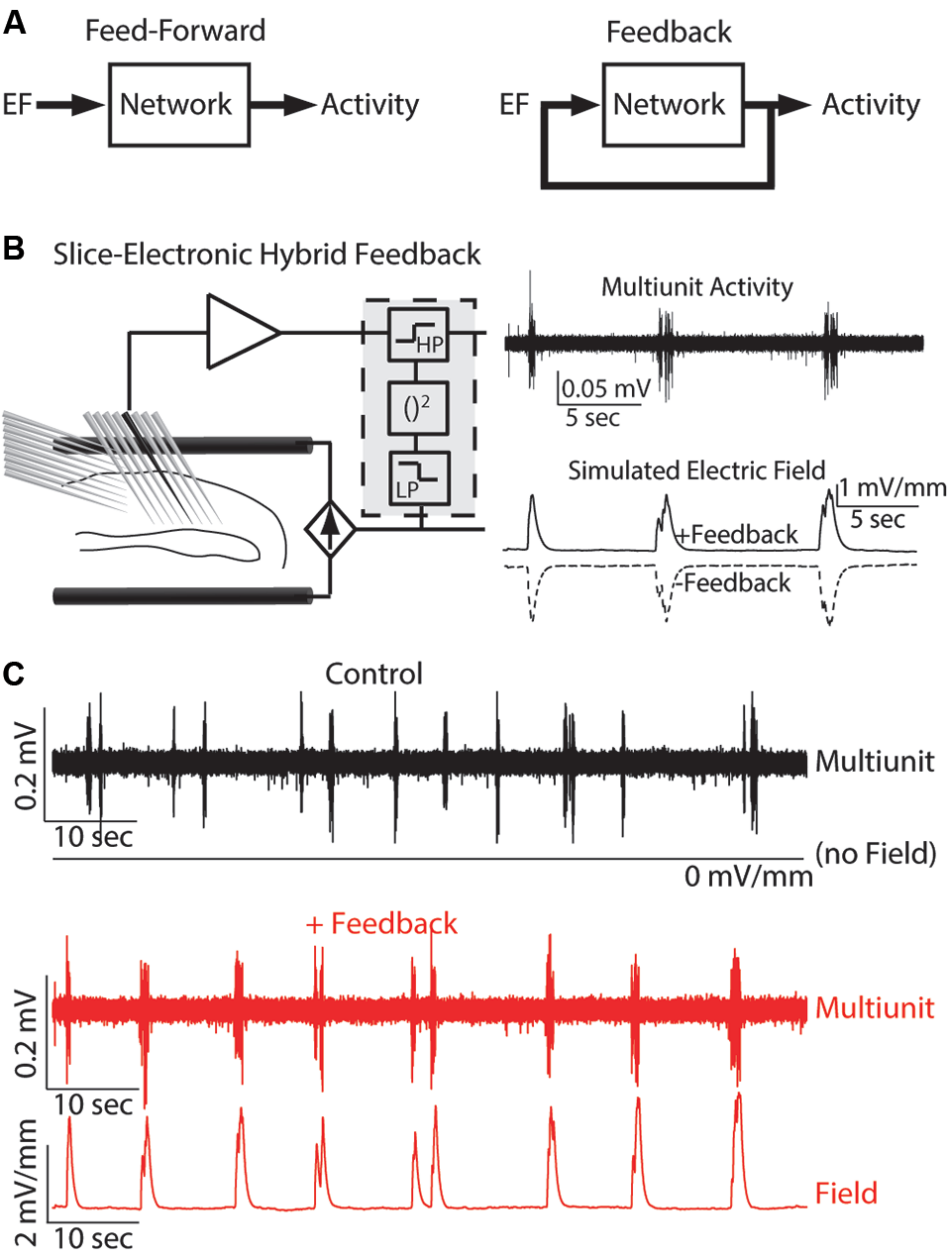
**Hybrid biological-electrical system.** **(A)** Control diagrams for both feed-forward and feedback application of EF stimulation. **(B)** Left: Schematic of the system where EF is applied based on the ongoing neuronal activity. Right: Example multiunit trace of typical endogenous activity (top) and the simulated EF applied for both positive and negative feedback. **(C)** Multiunit activity and applied EF for both control (top, black) and positive feedback (bottom, red). Reprinted with permission (Frohlich and McCormick, 2010).

We propose that understanding the effects of externally applied electric fields (EFs) requires mechanistic insight into the functional role of endogenous EFs that have been historically discounted as an epiphenomenon of cortical oscillations. Despite a number of studies (Francis et al., 2003; Deans et al., 2007; Radman et al., 2007) that have demonstrated the effect of weak EFs on rodent hippocampal networks *in vitro* (feed-forward stimulation with artificial waveforms such as sine-waves), a direct demonstration of a causal role of *endogenous* EFs in shaping cortical network dynamics has lacked. In particular, the open questions are: (1) if *naturalistic* EF waveforms have similar effects on network dynamics; (2) if the interaction dynamics differ between feed-forward and feedback application of EFs (Figure 1A); and (3) if neocortical areas, which typically exhibit lower cell densities, are equally sensitive to weak EFs. A recent study (Frohlich and McCormick, 2010) that leveraged the presence of spontaneous rhythmic activity in neocortical slices of ferrets addressed these questions. Indeed, EF waveforms that were previously recorded *in vivo* caused a pronounced enhancement of the spontaneous rhythmic activity in slices of visual cortex. Furthermore, the use of a hybrid biological-electronic system (Figure 1B) demonstrated that modulation of the endogenous electric field by real-time feedback stimulation altered the structure of spontaneous cortical oscillations in neocortex. Both positive and negative feedback stimulation were evaluated. In the case of positive feedback (Figure 1C), a depolarizing, activity-dependent EF computed in real-time from the multiunit activity was applied. This stimulation resulted in increased rhythmic structure of the spontaneous slow oscillation (measured by rhythmicity of UP states). However, when negative feedback was applied, the times between UP states exhibited greater variability resulting in decreased rhythmic structure. The positive feedback stimulation was designed to mimic the hypothesized interaction between endogenous EF and network activity *in vivo*. The enhancement and suppression of oscillatory structure with positive and negative feedback, respectively, therefore supports the conclusion that the endogenous EF causally modulates cortical network dynamics. These results not only propose endogenous EFs as a fundamental mechanism by which cortical synchronization is enhanced but also demonstrate the pronounced susceptibility of active cortical networks to weak EFs as provided by TCS.

Furthermore, the application of feedback EF waveforms also has potential as a novel class of brain stimulation therapeutics for the treatment of disorders of the central nervous system. Pioneering work on animal epilepsy models demonstrated the efficacy of such a non-pharmacological approach (Nakagawa and Durand, 1991; Schiff et al., 1994; Jerger and Schiff, 1995). These feasibility studies used similar hybrid systems where neural activity was recorded and a feedback stimulation signal computed and applied to a slice preparation. Seizure events are characterized by large amounts of highly synchronized network activity and may be modeled in tissue slices by elevation of extracellular concentration of potassium in the artificial cerebrospinal fluid (Frohlich et al., 2008). Seizure events are characterized by hyper-activity and thus hyperpolarizing neurons could be sufficient to reduce seizures by hyperactivity (Gluckman et al., 1996). However, the complex dynamics of neuronal networks caused hyperpolarizing DC stimulation to have only short-term effects in seizure suppression. In contrast, a hybrid system that applied EF stimulation based on the ongoing network dynamics (negative feedback) was able to suppress seizure-like events for up to 16 min (Gluckman et al., 2001). Translation to *in vivo* models has provided further support for the efficacy of such stimulation paradigms (Berenyi et al., 2012). This result highlights both the therapeutic possibilities of hybrid stimulation systems and the benefits of rational design of stimulation paradigms.

## Optimizing stimulation with large-scale computer simulations

**Figure 2:**
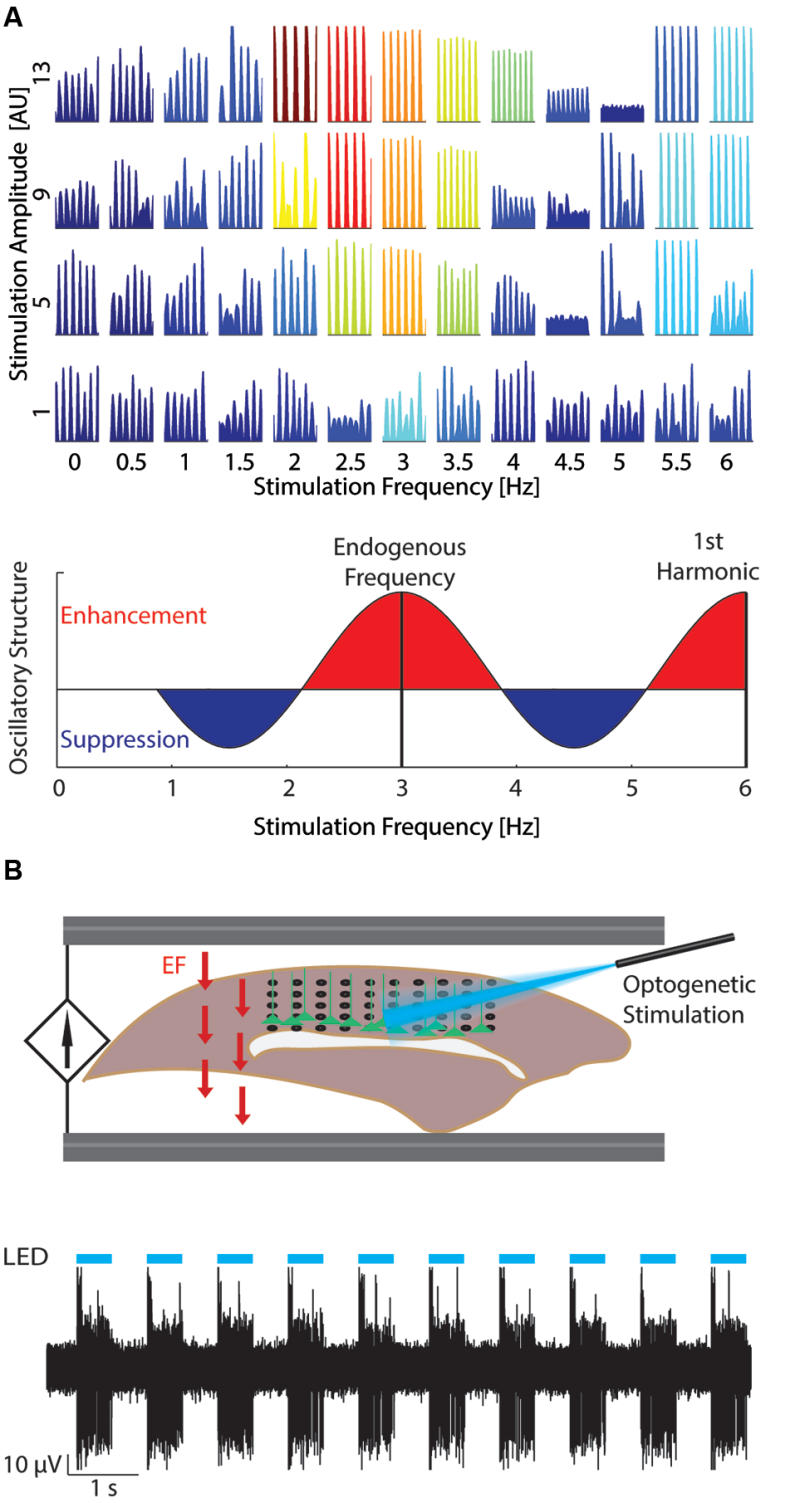
**Studying resonance dynamics with large-scale computational models and optogenetics.** **(A)** Top: Network response to varying stimulation amplitude (increasing bottom to top) and frequency (left to right). Color indicates power of network activity at the stimulation frequency. At low stimulation amplitudes, the network was most entrained by stimulation at the endogenous frequency (~3 Hz). Increased stimulation amplitude expanded the stimulation frequencies that entrained the network. Reprinted with permission (Ali et al., 2013). Bottom: Change in oscillatory structure for increasing tACS frequency. Red areas represent relative enhancement of oscillatory structure with maxima at the endogenous oscillation frequency and harmonics of the endogenous oscillation. Blue areas represent relative suppression with minima between harmonics of the endogenous oscillation. **(B)** Top: Experimental set-up (Schmidt et al., 2013). Optogenetic stimulation (blue) is applied to layer V pyramidal cells (green) from above to entrain the network at the desired frequency. EF (field arrows, red) is then applied through AgCl wires to model the effect of TCS. Neural data may then be recorded, for example with a multielectrode array pictured here (black). Bottom: Example multiunit trace (black) displaying strong entrainment to the optogenetic stimulation (cyan). Reprinted with permission (Schmidt et al., 2013).

Computer simulations are an important tool to investigate the interactions between endogenous oscillations and TCS. These simulations enable the study of network dynamics with single-cell resolution at the scale of millions of neurons by leveraging advances in parallel scientific computing and introduction of efficient models which retain the network-level accuracy of prior, computationally more expensive models (Izhikevich, 2004). In such simulations, the interaction between endogenous oscillations and TCS may be studied by applying a simulated EF to the model network (Reato et al., 2010, 2013; Ali et al., 2013). For example, one recent study from our group (Ali et al., 2013) contrasted the effects of both tACS and tDCS on an endogenously oscillating model network. The application of tACS at a stimulation frequency matched to the frequency of the endogenous oscillation enhanced the endogenous oscillation to a greater extent than tDCS. Importantly, networks exhibited the greatest enhancement when stimulated near the frequency of the endogenous oscillation (3 Hz), a sign of resonance dynamics (Figure 2A). In contrast, network activity was reduced when stimulation at a frequency (4.5 Hz) between the endogenous frequency and its first harmonic was applied. Therefore a key component of rational design of tACS is measuring the ongoing oscillations and matching the stimulation frequency accordingly.

The balance of excitatory and inhibitory synaptic activity is another important characteristic of neuronal networks that can be studied at the network level in simulations only. Inhibitory interneurons are less susceptible to changes in EF due to their small size compared to pyramidal neurons. However, inhibitory activity may be increased to balance increased excitatory activity caused by stimulation (Reato et al., 2010). Modulation of inhibitory activity by tACS was described in a computational model of a network that intrinsically oscillated at 25 Hz. The net firing rate of neurons did not change with low frequency tACS applied, however the temporal patterning was changed. Inhibitory activity increased at a greater rate than excitatory activity, which had a balancing effect on the firing rate. This study demonstrated a means by which tACS can modulate inhibitory activity, through indirect action on excitatory/inhibitory balance rather than by a direct modulation of membrane potential. Therefore, tACS modulation of excitatory/inhibitory balance may have applications in the treatment of autism and schizophrenia where the underlying cortical circuits exhibit abnormal excitatory/inhibitory balance (Rubenstein and Merzenich, 2003; Kehrer et al., 2008; Yizhar et al., 2011).

## Combining optogenetics and dynamic systems theory

Optogenetics is typically used to activate specific neural pathways, which allows examination of the underlying circuitry involved in different behaviors (Miesenbock and Kevrekidis, 2005; Gradinaru et al., 2007; Fenno et al., 2011). Yet, optogenetic stimulation can also be a valuable tool for entraining network activity in a wide range of frequencies. For example, slow-wave oscillations have been entrained using optogenetic stimulation of layer five (LV) pyramidal cells *in vivo* (Beltramo et al., 2013). The depolarizing action of optogenetic stimulation was sufficient to evoke UP states across the network in both LV and LII/III. Faster rhythms have also been entrained using optogenetic stimulation of fast-spiking inhibitory interneurons (Cardin et al., 2009). Indeed, *in vivo* optogenetic stimulation of varying frequencies caused the greatest effect on the rhythmic structure of the local field potential (LFP) when the stimulation frequency was between 40 and 50 Hz (Carlen et al., 2012). Isolated *in vitro* networks of cultured neurons may also be entrained with optogenetic stimulation (Pina-Crespo et al., 2012). *In vivo* cortical networks are nonlinear systems that exhibit ongoing rhythmic activity. Due to this nonlinearity, the response to TCS will likely be different based on the current state of activity. We here propose that interaction dynamics of TCS and endogenous activity can be studied with optogenetic stimulation to induce *in vivo*-like activity patterns. For example, a slow oscillation can be entrained using optogenetic stimulation *in vitro* and EF can be applied while the resulting modulation of activity by TCS is measured using whole-cell patch clamp, multiunit, or LFP recordings (Figure 2B; Schmidt et al., 2013).

## Translating fundamental concepts into brain stimulation therapeutics

The above discussed approaches enable fundamental insights into how active cortical networks respond to stimulation. In particular, the application of modern neuroscience tools provides the unique opportunity to understand how non-invasive brain stimulation with EFs can modulate cortical oscillations that are impaired in a broad range of disorders of the central nervous system, such as schizophrenia (Uhlhaas and Singer, 2012) and epilepsy (Bazhenov et al., 2008). However, it is important to recognize that several major obstacles remain before successful translation of these novel basics findings to the clinical realm. First, the proposed approaches have not yet been broadly applied to cortical oscillations of different frequencies and underlying generators. For example, modulation of alpha oscillations with TCS has been successfully demonstrated in humans (Zaehle et al., 2010; Neuling et al., 2013), but the underlying mechanism is likely different from the enhancement of slow cortical oscillations discussed here. Specifically, alpha oscillations likely emerge from the dynamic interaction of the thalamus and cortex, whereas slow cortical oscillations are considered to be mostly of cortical origin (Timofeev et al., 2000) (yet see Blethyn et al., 2006). To what extent there will be convergence on one fundamental mechanism that applies to rhythmic activity patterns with different generators remains unknown. Second, computational simulations and *in vitro* animal experiments do not consider the complexity of delivering EFs to the brain through multiple layers of tissue and bone. Furthermore, targeting of specific cortical locations is difficult in gyrencephalic brains due to the different neuronal orientation across gyri and sulci. Since the effect of EFs on the membrane voltage depends on the relative orientation of the field to the somato-dendritic axis of neurons (Tranchina and Nicholson, 1986), the evaluation of TCS in an intact animal model with a gyrencephalic brain is a further important step towards the development of novel therapeutic TCS paradigms in humans.

## Conclusion

Rational design of TCS requires an understanding of the interaction between endogenous EF and network activity and the interaction between network activity and stimulation EFs. Hybrid systems have both established the role of endogenous EFs and resulted in successful control of network activity. Recent studies with large-scale computer models have begun to mechanistically elucidate the interaction dynamics between endogenous network activity and EF stimulation. We further propose *in vivo* and *in vitro* studies that leverage optogenetic stimulation to first entrain network activity which allows the targeted study of these interaction dynamics. Together, these interdisciplinary approaches will provide a foundation for the rational design of TCS.

## Conflict of interest statement

The authors declare that the research was conducted in the absence of any commercial or financial relationships that could be construed as a potential conflict of interest.
